# A novel model based on interleukin 6 and insulin-like growth factor II for detection of hepatocellular carcinoma associated with hepatitis C virus

**DOI:** 10.1186/s43141-021-00262-8

**Published:** 2021-10-29

**Authors:** Mohamed M. Omran, Sara Mosaad, Tarek M. Emran, Fathy M. Eltaweel, Khaled Farid

**Affiliations:** 1 Chemistry Department, Faculty of Science, Helwan University, Ain Helwan, Cairo, 11795 Egypt; 2 Chemistry Department, Faculty of Science, Damietta University, New Damietta, Damietta Egypt; 3 Clinical Pathology Department, Faculty of Medicine, Al-Azhar University, New Damietta, Egypt; 4 Tropical Medicine Department, Faculty of Medicine, Mansoura University, Mansoura, Egypt

**Keywords:** Hepatocellular carcinoma, Diagnosis, Blood markers, Interleukin, Insulin-like growth factor 2

## Abstract

**Background:**

The coexistence of cirrhosis complicates the early detection of hepatocellular carcinoma (HCC). Thus, novel biomarkers for HCC early detection are needed urgently. Traditionally, HCC detection is carried out by evaluating alpha-fetoprotein (AFP) levels combined with imaging techniques. This work aimed to assess interleukin (IL-6) and insulin-like growth factor 2 (IGF 2) as possible HCC markers in comparison to AFP in patients with and without HCC.

**Results:**

ROC analysis showed that IGF2 had the highest area under the curve (AUC) for discriminating HCC from liver cirrhosis (0.86), followed by IL6 (0.82), AFP (0.72), and platelet count (0.6). A four-marker model was developed and discriminated HCC from liver cirrhosis with an AUC of 0.97. The best cut-off was 1.28, at which sensitivity and specificity were 90% and 85%, respectively. For small tumor (< 2 cm), the model had an AUC of 0.95 compared to AFP (0.72). Also, the model achieved perfect performance with AUC of 0.93, 0.94, and 0.95 for BCLC (0-A), CLIP (0-1), and Okuda (stage I), respectively, compared to AFP (AUC of 0.71, 0.69, and 0.67, respectively).

**Conclusions:**

The four markers may serve as a diagnostic model for HCC early stages and help overcome AFP poor sensitivity.

## Impact statement

Worldwide, hepatocellular carcinoma is the sixth most common cancer and the second leading cause of death. The current work aimed to evaluate interleukin and insulin-like growth factor 2 as possible HCC markers compared to alpha-fetoprotein (AFP).

## Background

Worldwide, HCC is the commonest aggressive liver cancer [1, 2]. It is the 4th common cancer in Egypt, and HCV is the most risk factor for cirrhosis and HCC development [3]. In many cancers, survival is significantly improved by early detection [4]. AFP is the most used biomarker for detecting HCC and the only biomarker that has been validated for clinical use but with limited sensitivity. However, AFP has higher sensitivity when used in combination with abdominal ultrasound (63% vs. 45% US alone) [5]. HCC behavior is heterogeneous, with carcinogenesis involving several genetic alterations, and this could explain the limited performance of a single biomarker. A model that includes multiple biomarkers encompassing heterogeneous pathways in carcinogenesis and clinical factors (sex, age, and etiology of liver disease) has been developed and validated to improve the HCC early detection [6]. Carcinogenesis is a complex system that includes stromal cells, endothelial cells, immune cells, cytokines, and growth factors [7]. Thrombocytopenia is used to monitor the development of HCC [8]. IL-6 production during inflammatory conditions and infections is induced via stimulation of cells by IL-1 or tumor necrosis factor (TNF)-α or stimulation of Toll-like receptors after binding of pathogenic patterns of microbes [9]. IL-6 has several roles in HCC including decrease HCC cell apoptosis, stimulate hepatic DNA synthesis, natural killer cell dysfunctions [10, 11]. IGF2 is produced in the liver in response to growth hormone. IGF2 has several roles in the development of liver cirrhosis and HCC through the promotion of hepatocyte proliferation [12]. The current study, aimed to evaluate IL-6 and IGF 2 as noninvasive HCC biomarkers compared to AFP.

## Methods

### Patients

Two hundred fifteen chronic hepatitis C patients were recruited between December 2018 and August 2019. Informed consent was signed by all patients in compliance with Helsinki Declaration. The first group included 155 patients with HCC. They were diagnosed according to American Association for the Study of Liver Diseases (AASLD) guidelines, based on AFP > 400 and the presence of hepatic focal lesion(s) detected by liver ultrasound and confirmed by triphasic computed tomography (CT) and/or dynamic magnetic resonance image (MRI). The second group included 60 patients with HCV-induced liver cirrhosis defined by clinical, biochemical, and imaging findings as splenomegaly. They were followed up for 6 months to ensure the absence of HCC. Patients with HCC were classified using three common staging systems: Barcelona Clinic Liver Cancer (BCLC), Cancer of Liver Italian Program (CLIP), and Okuda systems [13]. None of the patients with HCC previously underwent trans-arterial chemoembolization or radiofrequency ablation. The severity of chronic liver disease was determined using the Child-Pugh score. All patients were positive for HCV-antibody and HCV-RNA. Patients with chronic diseases (cardiovascular, renal, autoimmune, rheumatoid arthritis, or hepatitis B virus) were excluded. In addition, patients with other thrombocytopenia or HCC causes or have other malignancies were also excluded from the study.

### Laboratory investigation

Tri-potassium dihydrogen ethylenediaminetetraacetate was used as an anticoagulant and then platelet count was performed using an automated hematology analyzer (Micros 60; Horiba medical, Montpellier, France). International normalized ratio (INR) was performed using a special device (Coatron M1; TECO, Neufahrn, Germany). Routine liver function tests were done using serum samples and an automated biochemistry analyzer (BT1500; Biotecnica instruments S.P.A, Italy). Serum AFP and HCV antibodies were evaluated using an immune-fluorescence assay by auto-analyzer (Mini-Vidas; bioMérieux, Marcy L’Etoile, France). The presence of serum HCV-RNA was determined by quantitative real-time polymerase chain reaction (COBAS Ampliprep/COBAS TaqMan; Roche Diagnostics, Pleasanton). Serum interleukin 6 and insulin-like growth factor II levels (pg/ml) were ascertained using a quantitative sandwich enzyme immunoassay technique (Elabscience Biotechnology Co., Ltd., USA) according to the manufacturer’s instructions. The sensitivity of IL-6 was 7.0 pg/mL, and IGF II was 2.0 Pg/ml. Both intra-CV and inter-CV are < 10%. Laboratory investigators were blind to the clinical data of each examined sample.

### Statistical analyses

SPSS software (SPSS Inc.) version 25.0 was used for data analysis. Categorical data were expressed as numbers or percentages and compared using the Chi-square test or Fisher’s exact test, when appropriate. Continuous variables were expressed as means ± standard deviations (SD) or medians and ranges. The student’s *t* test or the non-parametric Mann–Whitney *U* test was used for comparing means between two groups. The ANOVA test or the non-parametric Kruskal–Wallis test was used for comparing means between more than two groups. Correlation test investigated the relation between each two variables among each group. ROC analyses were done for markers to differentiate between HCC and cirrhosis, and the best cut-off points and diagnostic indices were calculated. Multivariate linear regression analysis was performed for HCC prediction. All statistical tests were two-sided. *P* values less than 0.05 were considered significant.

## Results

### Clinical and laboratory data of study groups

More than half of the HCC patients were males (no. = 84; 54%) compared to more than one-third (*n* = 26; 43.3%) of liver cirrhosis patients, with no significant difference in age between the two groups. The laboratory and clinical data of the study groups are shown in Table 1. Levels of AST, ALT, AFP, IL6, and IGF2 were significantly higher, but platelet count was significantly lower in HCC compared to other studied groups. There were no significant differences in albumin, bilirubin, and prothrombin-INR. There was no correlation between candidate biomarkers but there was correlation between IL6 and other routine parameters in studied patients (bilirubin (*r* = 0.52, *p* < 0.0001); lymphocytes (*r* = 59, *p* = 0.005); monocytes (*r* = 0.63, *p* = 0.006); basophils (*r* = 0.86, *p* < 0.0001)).

**Table 1:** Clinical and laboratory data of the studied groups

Variables	Cirrhosis group (*n* = 60)	HCC group (*n* = 155)	*P* value
Age (year)	57.1 ± 8.3	59.4 ± 7.1	0.1
Gender			
Male no., %	26 (43%)	84 (54%)	0.17
Female no., %	34 (57%)	71 (46%)	
**Liver function tests**			
Alanine aminotransferase (U/L)	28 (22-40)	33 (24.5-64)	0.02
Aspartate aminotransferase (U/L)	31 (22-50)	40 (28.7-70)	0.049
Total bilirubin (mg/dl)	0.9 (0.7-1.2)	1.2 (0.8-1.9)	0.13
Albumin (g/L)	34.2 ± 7.1	33.6 ± 5.0	0.48
Prothrombin—INR	1.27 ± 0.2	1.34 ± 0.4	0.13
**Hematological tests**			
Platelet count (× 10^9^/L)	136 (77-171.5)	92.5 (61.5-153)	0.03
HCC tumor marker			
α—fetoprotein (U/L)	11 (5.5-18)	65.9 (7.8-514)	0.03
Interleukin 6 (pg/ml)	10.3 (7-18.8)	18.9 (12.4-45.1)	< 0.001
Insulin-like growth factor II (pg/ml)	2.6 (2-10)	34 (11-306.5)	0.001
Child-Pugh score; *n* (%)			
Child A	30 (50%)	120 (77.4%)	< 0.0001
Child B	30 (50%)	35 (22.6%)	

### The diagnostic performances of the single markers

The biomarkers diagnostic performance is illustrated in Table 2 according to the AUC. AFP showed an AUC of 0.72. The best cut-off point was 400 U/l with 27% sensitivity and absolute specificity. IGF-2 was the most powerful diagnostic marker with the highest AUC (0.86), followed by IL6 (0.82) and platelet count (0.6). The diagnostic power of single markers in relation with morphological features and staging systems of HCC classification are presented in Tables 3, 4, 5, and 6.

**Table 2:** Diagnostic power of investigated biomarkers and model for differentiating  liver cirrhosis vs. HCC

Markers	AUC (95% Cl)	Cut-off	Sensitivity	Specificity	PPV	NPV	Accuracy
IGF2	0.86 (0.78-0.94)	10	80	73.3	80	73	77
IL6	0.82 (0.66-0.89)	13.5	72	70	73.5	75	74.2
AFP	0.72 (0.65-0.8)	400	27	100	100	35	48
PLT	0.6 (0.51-0.96)	95	65	52	71	78	60
^a^Model	0.97 (0.92-1)	1.28	90	85	82	92	87

^a^A four-marker model (IGF2 + IL6 + AFP + PLT)

**Table 3:** Diagnostic power of AFP to discriminate patients with HCC from patients with cirrhosis in different three staging systems

Classification	AUC	Sens%	Spe%	PPV%	NPV%	AC%
**Number of nodules (*n*, %)**						
Single (67, 43.2%)	0.71	22	100	100	53	58
Multiple (88, 56.8)	0.74	31	100	100	52	60
**Macrovascular invasion (*n*, %)**						
Absent (141, 91%)	0.74	25	100	100	38	48
Present (14, 9%)	0.65	50	100	100	90	91
**Size of nodules (*n*, %)**						
<2 (48, 31%)	0.72	13	100	100	63	65
>2 (107, 69%)	0.75	33	100	100	49	59
**BCLC stage (*n*, %)**						
0-A (109, 70.3%)	0.71	19	100	100	41	48
B (46, 29.7)	0.8	52	100	100	75	80
**CLIP stage (*n*, %)**						
0-1 (122, 79%)	0.69	17	100	100	39	45
2-3 (33, 21%)	0.88	63	100	100	82	86
**Okuda stage (*n*, %)**						
Stage I (117, 75.5%)	0.67	23	100	100	41	50
Stage II (38, 24.5%)	0.89	39	100	100	70	75

**Table 4:** Diagnostic power of platelet count to discriminate patients with HCC from patients with cirrhosis in different three staging systems

Classification	AUC	Sens%	Spe%	PPV%	NPV%	AC%
**Number of nodules (*n*, %)**						
Single (67, 43.2%)	0.64	76	52	57	72	63
Multiple (88, 56.8)	0.58	57	52	50	58	54
**Macrovascular invasion (*n*, %)**						
Absent (141, 91%)	0.61	66	52	68	49	60
Present (14, 9%)	0.66	75	52	17	94	54
**Size of nodules (*n*, %)**						
<2 (48, 31%)	0.57	62	52	38	74	55
>2 (107, 69%)	0.63	66	52	60	59	59
**BCLC stage (*n*, %)**						
0-A (109, 70.3%)	0.61	67	52	63	56	60
B (46, 29.7)	0.6	62	52	38	74	55
**CLIP stage (*n*, %)**						
0-1 (122, 79%)	0.62	68	52	64	55	61
2-3 (33, 21%)	0.6	60	52	34	76	55
**Okuda stage (*n*, %)**						
Stage I (117, 75.5%)	0.65	71	52	65	59	63
Stage II (38, 24.5%)	0.56	55	52	37	69	53

**Table 5:** Diagnostic power of IL6 to discriminate patients with HCC from patients with cirrhosis in different three staging systems

Classification	AUC	Sens%	Spe%	PPV%	NPV%	AC%
**Number of nodules (*n*, %)**						
Single (67, 43.2%)	0.82	78	70	44	91	72
Multiple (88, 56.8)	0.82	78	70	67	81	74
**Macrovascular invasion (*n*, %)**						
Absent (141, 91%)	0.81	77	70	67	78	73
Present (14, 9%)	0.85	83	70	36	96	72
**Size of nodules (*n*, %)**						
<2 (48, 31%)	0.82	78	70	44	91	72
>2 (107, 69%)	0.82	78	70	67	81	74
**BCLC stage (*n*, %)**						
0-A (109, 70.3%)	0.77	71	70	63	78	71
B (46, 29.7)	0.91	91	70	53	96	76
**CLIP stage (*n*, %)**						
0-1 (122, 79%)	0.78	75	70	67	78	72
2-3 (33, 21%)	0.95	88	70	44	96	74
**Okuda stage (*n*, %)**						
Stage I (117, 75.5%)	0.79	77	70	65	81	73
Stage II (38, 24.5%)	0.88	80	70	47	85	73

**Table 6:** Diagnostic power of IGF2 to discriminate patients with HCC from patients with cirrhosis in different three staging systems

Classification	AUC	Sens%	Spe%	PPV%	NPV%	AC%
**Number of nodules (*n*, %)**						
Single (67, 43.2%)	0.90	88	73.3	67	92	80
Multiple (88, 56.8)	0.86	79	73.3	73	82	78
**Macrovascular invasion (*n*, %)**						
Absent (141, 91%)	0.88	83	73.3	81	79	80
Present (14, 9%)	0.88	80	73.3	36	96	77
**Size of nodules (*n*, %)**						
<2 (48, 31%)	0.85	75	73.3	56	89	76
>2 (107, 69%)	0.89	86	73.3	77	85	81
**BCLC stage (*n*, %)**						
0-A (109, 70.3%)	0.88	77	73.3	77	79	77
B (46, 29.7)	0.88	79	73.3	77	88	77
**CLIP stage (*n*, %)**						
0-1 (122, 79%)	0.88	84	73.3	77	82	80
2-3 (33, 21%)	0.89	78	73.3	77	92	77
**Okuda stage (*n*, %)**						
Stage I (117, 75.5%)	0.89	87	73.3	79	85	82
Stage II (38, 24.5%)	0.89	79	73.3	61	89	77

#### Development of a novel model for HCC diagnosis

According to the current results, AFP had a poor diagnostic performance, and there was a limited diagnostic value of a single biomarker because of HCC biological heterogeneity. Furthermore, there were no correlations between the biomarkers. All these data imply that each biomarker provides distinct information. It was anticipated that the diagnostic performance would improve if individual potential biomarkers (AFP, IGF2, IL6, and platelet count) were combined and yielded a single model with optimal diagnostic performance. This combination was performed using multivariate discriminate analysis (MDA) for predicting HCC patients as follows; MDA model = 1.17 + AFP (U/L) × 0.002 + IGF2 (pg/ml) × 0.001 + IL6 (pg/ml) × 0.008 – platelet count (× 10^9^/L) × 0.001). The median and range of the model were 2.27 (1.65-2.9) and 1.19 (1.14-1.22) in HCC and liver cirrhosis patients, respectively (*p* < 0.001). Using ROC analysis, the model showed an AUC of 0.97, which is higher than that of AFP (0.72), indicating better diagnostic performance. The best cut-off was 1.28, at which sensitivity and specificity were 90% and 85%, respectively (Table 2). ROC analysis showed a good performance of the model in patients with single nodule, absent macrovascular invasion, and small tumor size < 2 cm (AUC of 0.95, 0.94, and 0.95, respectively), compared to AFP (AUC of 0.71, 0.74, and 0.72, respectively). When applying the three staging systems for HCC classification and then performing ROC analysis, the model achieved perfect performance with AUC of 0.93, 0.94, and 0.95 for BCLC (0-A), CLIP (0-1), and Okuda (stage I), respectively, compared to AFP (AUC of 0.71, 0.69, and 0.67, respectively) (Table 7).

**Table 7:** Performance of the four-marker model (IGF2+ IL6+ AFP+ platelet count) according to the BCLC, CLIP, and Okuda staging systems

Classification	AUC (95% Cl)	Sens%	Spe%	PPV%	NPV%	AC%
**Number of nodules (*n*, %)**						
Single (67, 43.2%)	0.95 (0.89-1.0)	90	85	82	92	87
Multiple (88, 56.8)	0.95 (0.88-1.0)	90	85	82	92	87
**Macro vascular invasion (*n*, %)**						
Absent (141, 91%)	0.94 (0.86-1.0)	88	85	88	85	86
Present (14, 9%)	1.0 (1.0-1.0)	100	85	67	100	88
**Size of nodules (*n*, %)**						
< 2 (48, 31%)	0.95 (0.87-1.0)	92	85	85	92	88
> 2 (107, 69%)	0.98 (0.93-1.0)	94	85	88	92	99
**BCLC stage (*n*, %)**						
0-A (109, 70.3%)	0.93 (0.96-1.0)	83	85	83	85	84
B (46, 29.7)	0.99 (0.96-1.0)	100	85	80	100	91
**CLIP stage (*n*, %)**						
0-1 (122, 79%)	0.94 (0.85-1.0)	87	85	87	85	86
2-3 (33, 21%)	1.0 (1.0-1.0)	100	85	71	100	89
**Okuda stage (*n*, %)**						
Stage I (117, 75.5%)	0.95 (0.88-1.0)	92	85	86	92	89
Stage II (38, 24.5%)	0.96 (0.87-1.0)	86	85	75	92	85

## Discussion

Various interleukins and growth factors were reported to be generated by hepatoma tissue and secreted in the blood [14, 15]. HCC is an inflammatory-related tumor modulated by JAK/STAT signaling pathway and immune/inflammatory response [16]. JAK/STAT signaling pathway was activated by different cytokines and growth factors such as interferon, interleukin, and IGF, which bind to their respective transmembrane receptors [17]. HCC is being a biologically heterogeneous tumor, which clarifies the single biomarker performance limitation [18, 19]. IL6, as a pro-inflammatory mediator, was reported to prominently increase in hepatic inflammation, viral infection, and HCC [20, 21]. IL6 had specificity and sensitivity of 93% and 77% compared with AFP (41% and 65%) [10]. In the current study, IL-6 had a sensitivity and specificity of 78% and 70% compared with AFP (27% and 100%) for HCC diagnosis. The liver is the main organ in IGF2 production. According to many studies, IGF2 levels decrease in cirrhosis, compared to normal individuals and increase in HCC [12]. Levels of IGF2 messenger RNA and protein increase > 20-fold in 15% of human HCC tissues compared with non-tumor liver tissues [22]. In the current study, the IGF2 level was significantly higher in HCC patients than in cirrhotic patients. The IGF2 had a sensitivity of 80%, and specificity of 73.3% in differentiating HCC from cirrhosis. According to Aleem et al., the sensitivity and specificity were 75% and 60% [23]. Another study reported a sensitivity and specificity of 73% and 64% [24], while a third study found a sensitivity and specificity of 22% and 91% [25]. These differences in the results may be due to differences in the inclusion criteria or clinical data of the studied population or different etiology (HCV and/or HBV). Thrombocytopenia was present in about two-thirds of HCC patients (60.4%) [26]. In the current study, the platelet count, had an AUC of 0.6, sensitivity of 65%, and specificity of 52% for diagnosis of HCC. This is in line with Chern-Horng Lee who reported a platelet count AUC of 0.64 [27, 28]. The combination of AFP, IGF2, IL6, and platelet count outperformed a single marker in terms of diagnostic performance. AUC increased to 0.97, with 90% sensitivity and 85% specificity. Furthermore, this model showed an excellent performance in patients with a single nodule (0.95 AUC and 90% sensitivity) and tumor size < 2 cm (0.95 AUC and 92% sensitivity). In contrast, alpha-fetoprotein routine test (HCC-ART) and simplified HCC-ART score had sensitivities of 70% and 82%, respectively, in diagnosing small-size HCC [29]. The combination of AFP and Des-gamma-carboxyprothrombin yields an AUC of 0.846 [30]. Omran et al. reported that the combination of acid glycoprotein, albumin, C reactive protein, and AFP has an AUC of 0.92 and a sensitivity of 85% [31]. According to Attallah et al., combining cytokeratin-1 and nuclear protein-52 with AFP provides an AUC, sensitivity, and specificity of 0.90, 80%, and 92%, respectively, for identifying HCC [32]. The combination of osteopontin (OPN) and AFP increases the AUC to 0.88. Also, the combination of OPN and liver enzymes increases the AUC to 0.87 [33]. In contrast, the combination of AFP and ultrasound has a low sensitivity of 63% for early HCC diagnosis. Furthermore, the sensitivities of CT and MRI in predicting early HCC are 62.5% and 83.7%, respectively [5].

## Conclusion

The current study provided a four-marker model (IGF2, IL6, PLT, and AFP) that could improve the early diagnosis of HCC.

## Data Availability

Authors declare that all generated and analyzed data are included in the article.

## Abbreviations

AC: Accuracy
AFP: Alpha-fetoprotein
HCC-ART: Alpha-fetoprotein routine test
AUC: Area under ROC curve
AASLD: Association for the Study of Liver Diseases
BCLC: Barcelona Clinic Liver Cancer
CLIP: Cancer of Liver Italian Program
CT: Computed tomography
HCC: Hepatocellular carcinoma
IGF 2: Insulin-like growth factor 2
IL-6: Interleukin
MRI: Magnetic resonance image
MDA: Multivariate discriminate analysis
NPV: Negative predictive value
OPN: Osteopontin
PPV: Positive predictive value
SD: Standard deviation
Sen: Sensitivity
Spe: Specificity
SPSS: Statistical package for social science
